# Central Diabetes Insipidus Masked by Uncontrolled Diabetes Mellitus: A Challenging Case Managed With Indapamide

**DOI:** 10.7759/cureus.21897

**Published:** 2022-02-04

**Authors:** Eyleen Gonzalez, Lorena Nuñez, Yavelkis Perez, Indira Atencio, Alex Pineda, Myron Miller, Stanley M Chen Cardenas

**Affiliations:** 1 Medicine, Facultad de Medicina, Universidad de Panama, Panama, PAN; 2 Internal Medicine, Hospital Nicolas A. Solano, West Panama, PAN; 3 Medicine, Sinai Hospital of Baltimore, Baltimore, USA; 4 Endocrinology, Diabetes and Metabolism, Johns Hopkins University School of Medicine, Baltimore, USA

**Keywords:** desmopressin, diabetic ketoacidosis (dka), indapamide, thiazides, traumatic brain injury, hypernatremia, polyuria, diabetes insipidus

## Abstract

A 44-year-old man with a history of traumatic brain injury (TBI) presented to the emergency room (ER) with diabetic ketoacidosis (DKA). After resolution of DKA, the patient had persistent polyuria (up to 5.5 L/24 h) associated with low specific gravity (1.002-1.005) and severe hypernatremia (up to 186 mmol/L) that led us to consider the possibility of central diabetes insipidus (DI). Due to the lack of desmopressin availability in our country, we managed the patient using indapamide. Polydipsia and polyuria in a patient with controlled diabetes mellitus (DM) should raise suspicion for alternative etiologies, including DI. Appropriate fluid management during hospitalization is critical to avoid life-threatening complications. TBI is an important cause of central DI and should be treated with desmopressin, an arginine-vasopressin (AVP) analog. In the absence of desmopressin, alternative options can help patients with central DI, including thiazides, carbamazepine, chlorpropamide, among others less studied.

## Introduction

Diabetic ketoacidosis (DKA) is a life-threatening complication of diabetes mellitus (DM) [1]. It occurs more commonly in type I DM but can present in type II DM when the insulin deficit is marked. DKA is characterized by polydipsia, polyuria, altered mental status, acidosis with electrolyte disturbances such as hyponatremia, pseudohyponatremia, or hypernatremia. Diabetes insipidus (DI) in contrast, is a disorder of water balance that occurs much less frequently than DM. DI can be classified as central when the problem is in the hypothalamic-pituitary region with insufficient production of arginine-vasopressin (AVP) and nephrogenic when the kidney does not respond to AVP [2-3]. Both forms of DI lead to a decrease in water reabsorption in the kidney resulting in hypo-osmolar polyuria. In the absence of thirst or fluid access, DI can lead to severe dehydration, hypernatremia, and death.

In both DKA and DI, polyuria and hypernatremia can occur, which can be challenging to manage if they co-exist. We present a patient admitted with DKA who had persistent polyuria and hypernatremia despite correction of hyperglycemia. Due to the lack of desmopressin treatment for central DI in our low-resource healthcare setting, an inexpensive alternative medication resulted in successful treatment. 

## Case presentation

A 44-year-old man with a history of a traumatic brain injury (TBI) after being hit by a car as a pedestrian suffered a left frontal and orbital fracture 11 months prior to presentation. He presented to the emergency room (ER) with one week of generalized weakness, worsening polyuria, polydipsia, and altered mental status. He was found to have DKA with a blood pH of 7.2, bicarbonate of 5.9 mmol/L, carbon dioxide 15 mmHg, anion gap 25, glucose 484 g/dL, positive urinary ketones, and normal lactate. He was managed according to the DKA protocol. 

**Figure 1:**
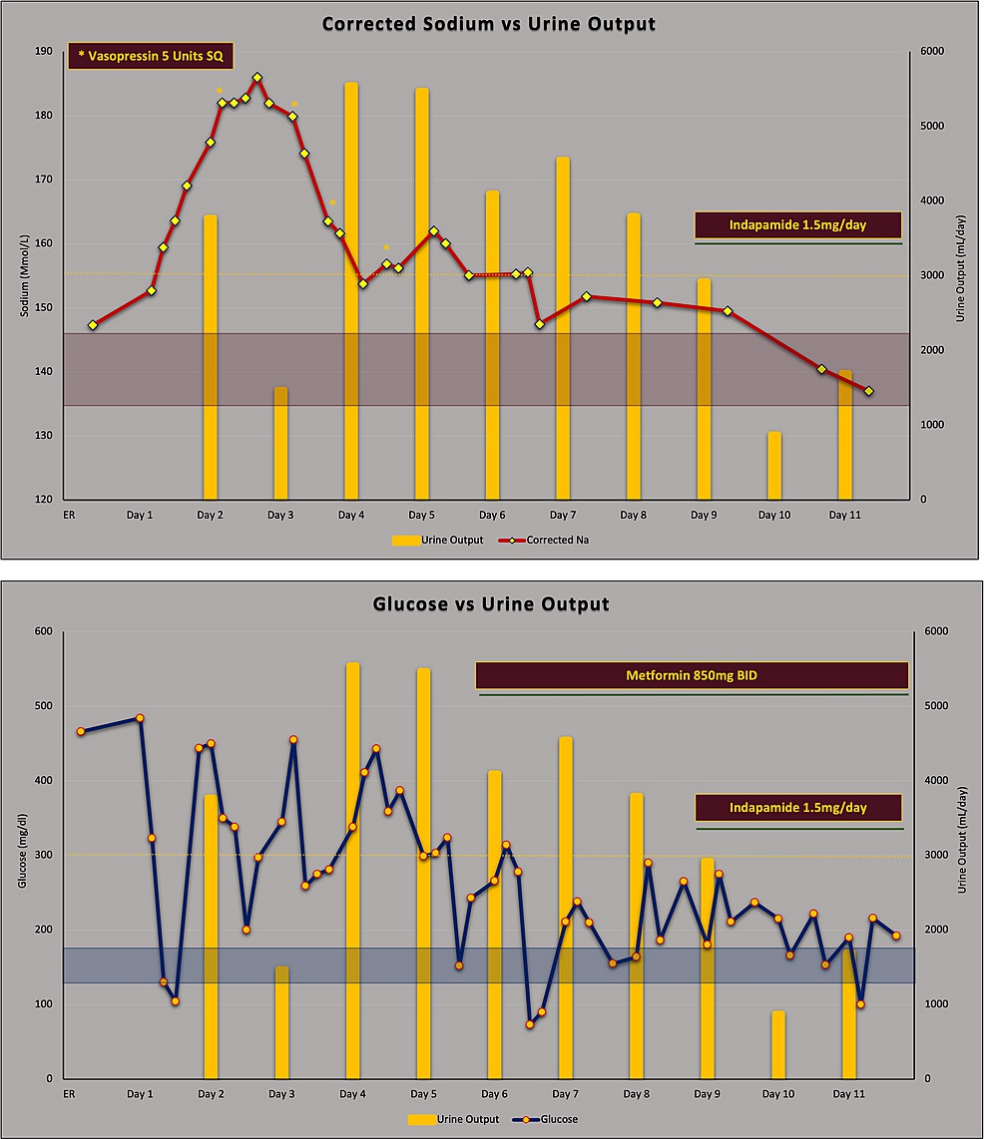
Corrected sodium, glucose, and urine output. Description of the relationship between corrected sodium [formula: corrected sodium = measured Na + [(glucose level - 100) x 0.016] and urine output (top). Relationship between glucose and urine output (bottom) associated with medications used in the patient (vasopressin, metformin, and indapamide).

Over the next 24 h, his serum glucose was challenging to control, requiring up to 14 units of insulin per hour while fasting. At the same time despite persistent hyperglycemia, his serum sodium continued rising reaching a maximum corrected of 186 mmol/L (Figure 1). After DKA resolution, hypernatremia and excessive urination persisted (>3L/day). Initially, it was thought that polyuria was due to glycosuria from uncontrolled diabetes. However, its persistence after glucose was <180 mg/dL, led us to consider alternative explanations. On reviewing his course, increased serum sodium level occurred rapidly in the setting of limited access to water and high urine output (>400 mL/h) after DKA resolution. The specific gravity in his initial urinalysis was 1.020 with corrected serum sodium of 147 mmol/L. Over the following day, the specific gravity decreased to 1.005 while corrected serum sodium reached 176 mmol/L. Given the history of TBI and clinical findings (Table 1) we considered the possibility of central DI. Our hospital unfortunately did not have the ability to measure urine osmolality or perform a pituitary MRI at the time. His calculated water deficit was up to 14 L and hypernatremia developed in <48 h. An aggressive hypotonic fluid replacement was started and as soon as the patient was awake he drank free water in response to his intact thirst mechanism. Additionally, given his progressive increase in urine output and severe hypernatremia, a total of four doses of five units sub-cutaneous (SQ) of vasopressin were administered during days two to four of admission resulting in a reduction in diuresis to as low as 75 mL/h, thus excluding the possibility of nephrogenic DI (Figure 1). The hospital course was complicated by transient electrolyte abnormalities such as hypokalemia, hypophosphatemia, and acute kidney injury (AKI) (Table 1). By day five to seven of admission, blood glucose was controlled and no DKA precipitant was found. He was fully awake and drinking large amounts of water to maintain the fluid balance close to neutral while serum sodium in the 150 s. 

**Table 1:** Laboratories on admission and discharge. *Obtained two days prior to discharge with a sodium 146 mmol/L BUN, blood urea nitrogen

Laboratory	Reference range	Admission	Discharge
Sodium (mmol/L)	135-145	140	137
Glucose (mg/dL)	70-100	466	185
Corrected sodium	135-145	147	138
Serum osmolality (mOsm/L)	275-295	314	288
Creatinine (mg/dL)	0.7-1.3	1.06	0.7
BUN (mg/dL)	8-20	23	6
Potassium (mmol/L)	3.5-5.1	3.8	3.5
Bicarbonate (mmol/L)	22-30	5.9	28.9
Anion gap (mmol/L)	8-16	25	3
Calcium (mg/dL)	8.6-10.3	10.3	9.6
White blood cells (10^3^/uL)	4-10	10.3	6.7
Neutrophils (%)	50-80	81.2	59.6
Hemoglobin (g/dL)	13-17	18.2	15.3
Hematocrit (%)	39-50	52.6	42.4
Platelets (10^3^/uL)	140-400	182	242
Urine specific gravity	1.000-1.030	1.020	1.003*
Glycosuria	Negative	4+ (>1000 mg/dL)	Negative*
Urine ketones	Negative	3+	Negative*

On planning for discharge, we found that desmopressin was not available in the country. Based on its efficacy in this setting, safety profile, accessibility, and low cost, we started treatment with indapamide 1.5 mg daily. Even though the patient was normotensive, he tolerated the drug well except for mild hypokalemia. His urine output decreased markedly from up to 5.5 L after DKA resolution to as low as 0.9 L per day by one day after starting indapamide (Figure 1). Serum sodium level decreased to 137 mmol/L, specific gravity remained low at 1.003 while drinking water as per thirst. He was successfully discharged with insulin glargine 36 units/day, lispro 12 units before meals, metformin 850 mg BID, indapamide 1.5 mg/day, and oral potassium supplements. 

On further questioning, he recalled that after the motor vehicle accident he was always thirsty and urinating large volumes at home that got progressively worse until the current hospitalization. He was discharged with plans for follow-up evaluation for other potential hypothalamic-pituitary hormonal deficiencies from the TBI. The patient was involved in the decision-making process and was very pleased with the results. Three weeks after discharge, he reported a resolution of polyuria and a normal water intake.

## Discussion

It is important to keep a broad differential diagnosis in patients with polyuria, particularly when unusual clinical features are present. In this young patient, severe dehydration evidenced by AKI, hemoconcentration, and hypernatremia was associated with high instead of low urine output that persisted after DKA resolution. Polyuria can be due to solute diuresis where urine osmolality (Uosm) is >300 mOsm/kg and can occur during hyperglycemia, recovery from acute renal failure, relief of urinary obstruction or exogenous solute load from enteral or parenteral nutrition, radiocontrast dyes, sodium, or mannitol. Polyuria can also result from water diuresis where Uosm is < 100 mOsm/kg and is caused by central or nephrogenic DI. A combination of both solute and water diuresis (mixed polyuria) can develop with Uosm between 100 and 300 mOsm/kg and could be due to partial DI or multifactorial [4-5]. The diuresis during uncontrolled DM is osmotic and independent from AVP; in fact, in DKA, AVP is elevated, granted that hypovolemia and nausea/vomiting are often present [6-7]. In our patient, the combination of hyperglycemia and central DI explains his initial excessive urination. After controlling hyperglycemia, central DI emerged as a more likely cause. 

Desmopressin was unavailable, which allowed us to search for alternative treatments for partial central DI. Central DI is routinely treated with oral desamino-D-arginine-8-vasopresssin (DDAVP or desmopressin), a longer-acting and V2 receptor-specific AVP analog. This drug can also be prescribed as sublingual, intranasal, inhaled, subcutaneous, or IV. Prior to the availability of DDAVP in the late 1970s, alternative drugs such as chlorpropamide, carbamazepine, and thiazides were studied. Chlorpropamide is a long-acting first-generation sulfonylurea previously used to treat DM. Its antidiuretic properties were discovered in the 1960s when a patient with DI took the medication by mistake [8]. Chlorpropamide can increase AVP secretion and enhance its action in the kidney [9-10]. It is effective in reducing urine output and increasing urine osmolality in patients with DI. Doses between 125 and 1000 mg/day have been used, but the risk of hypoglycemia is the limiting factor in using this drug [8, 11]. Other sulfonylureas lack this antidiuretic effect. Also, metformin has reported antidiuretic effects [12]. It is possible that giving metformin to our patient contributed to his improvement. Carbamazepine and oxcarbazepine are voltage-gated sodium channel blockers used as antiepileptics. It appears that they can increase AVP secretion. More recently, direct action on the V2R-G-protein complex with increased AQ2 expression and resultant decreased water excretion independent from AVP has been described [13]. Prolongation of AVP half-life and resetting the AVP-osmotic hypothalamic threshold are potential mechanisms. Reports using carbamazepine in doses of 200-600 mg/day and 600-900 mg BID for oxcarbazepine have been documented [10, 14]. Thiazides are commonly used diuretics that block the sodium-chloride transporter in the distal tubule. Hydrochlorothiazide, indapamide, and chlorothiazide have been studied in DI [15]. Thiazides increase sodium excretion leading to volume contraction, reduction in glomerular filtration rate, increasing proximal sodium, and water reabsorption. It is believed that this results in less solute and water delivery to the distal tubules and collecting ducts reducing the urine output, therefore, AVP independent [16]. A study using indapamide demonstrated a reduction in urine output of about 40% together with an increase in urine and a decrease in serum osmolality comparable to chlorpropamide [17]. Non-steroidal anti-inflammatory drugs (NSAIDs) such as ibuprofen and indomethacin have shown a reduction in urine volume. Prostaglandin E2 (PGE2) is an inhibitor of the AVP effect, as well as a local vasodilator on the renal vasculature, therefore, inhibition of PGE2 by NSAIDs has an antidiuretic effect [10, 18]. Clofibrate has also reported antidiuretic properties possibly by increasing the release of AVP [19]. Furthermore, acetaminophen, amitriptyline, vincristine, cyclophosphamide, diazoxide, valproic acid, selective serotonin reuptake inhibitors (SSRIs), and selective serotonin reuptake inhibitors (SNRIs) have reported antidiuretic effects and can produce the syndrome of inappropriate antidiuretic hormone secretion [12].

We decided to use indapamide given its safety profile and availability in our institution [17]. Possibly this patient could maintain acceptable serum sodium levels with aggressive oral intake. However, if access to water is limited or severe illness is present, this may not be possible.

## Conclusions

We consider it is essential for the clinician to know that for central DI when DDAVP is not available or the cost is a problem, other alternatives can be used to lower urine output, stimulate AVP release, or potentiate its renal effect. This is particularly relevant in low-resource healthcare settings. Polyuria and hypernatremia should raise suspicion for DI, even in patients with uncontrolled DM, particularly when a risk factor such as TBI is present, as in this case. The coexistence of DKA and DI is rare and could be potentially lethal if not properly recognized and managed.
